# A Rare Association of Disseminated Granuloma Annulare With Recurrent Uveitis

**DOI:** 10.7759/cureus.53570

**Published:** 2024-02-04

**Authors:** Anca Cojocaru, Alexandra Maria Dorobanțu, Beatrice Bălăceanu, Irina Tudose, Olguța Anca Orzan

**Affiliations:** 1 Department of Dermatology, Elias Emergency University Hospital, Bucharest, ROU; 2 Department of Pathology, Elias Emergency University Hospital, Bucharest, ROU; 3 Department of Dermatology, Carol Davila University of Medicine and Pharmacy, Elias Emergency University Hospital, Bucharest, ROU

**Keywords:** non-infectious uveitis, granulomatous dermatitis, generalized granuloma annulare, chronic uveitis, granuloma annulare

## Abstract

Granuloma annulare is a benign chronic inflammatory granulomatous dermatosis with a variable clinical presentation. The disseminated form of the disease is characterized by a widespread papular eruption, primarily affecting the trunk, neck, and extremities. The development of granuloma annulare in patients with systemic diseases, such as diabetes mellitus, malignancy, or dyslipidemia, has been extensively documented. Still, only a few cases of granuloma annulare associated with recurrent uveitis have been reported. Herein, we present a rare case of generalized granuloma annulare that was associated with concomitant recurrent uveitis in a 60-year-old male patient with a history of type II diabetes mellitus. A general physical exam revealed widespread erythematous papules in an annular pattern on the trunk, characteristic of granuloma annulare. A series of tests were conducted, including autoimmune workup, all within normal limits. Histopathologic findings revealed features consistent with granuloma annulare. The patient was successfully treated with systemic corticosteroids for the uveitis and isotretinoin for the skin lesions. A close follow-up is recommended given the rare association of granuloma annulare and uveitis.

## Introduction

Granuloma annulare (GA) is a chronic, self-limiting, inflammatory, and granulomatous skin disease of unknown etiology that affects both adults and children [1]. The disease presents with variable clinical features and is considered a benign condition. GA is more common among women, with a female-to-male ratio of 3:1 [2]. It is characterized by the development of erythematous or skin-colored papules that coalesce to form oval or ring-shaped lesions. GA can either be localized (localized GA) or disseminated (generalized GA). The disseminated form of GA is defined as a widespread eruption that frequently involves the trunk, neck, and extremities, which are sites of predilection for the localized variant [1].

It is important to acknowledge the considerable variation in the clinical presentation of GA among individuals; thus, the diagnosis is often made based on its characteristic histological features rather than its clinical appearance alone. GA is a typical non-infectious necrobiotic granulomatous reaction pattern that correlates with several different but relatively specific clinical presentations [3]. It is essential to differentiate GA from other dermatological conditions, such as sarcoidosis, which exhibits a mononuclear histiocytic cellular reaction but has an unknown pathogenesis [4,5].

GA can be triggered by various causes, such as diabetes mellitus, dyslipidemia, trauma, medications, vaccinations, or viral infections including SARS-COV-2 [6]. Additionally, it has been associated with malignancies, such as prostate carcinoma and bladder neoplasms [7].

The development of GA in patients with systemic diseases has been extensively documented. However, few cases have been reported where an association with recurrent uveitis has been observed.

## Case presentation

**Figure 1:**
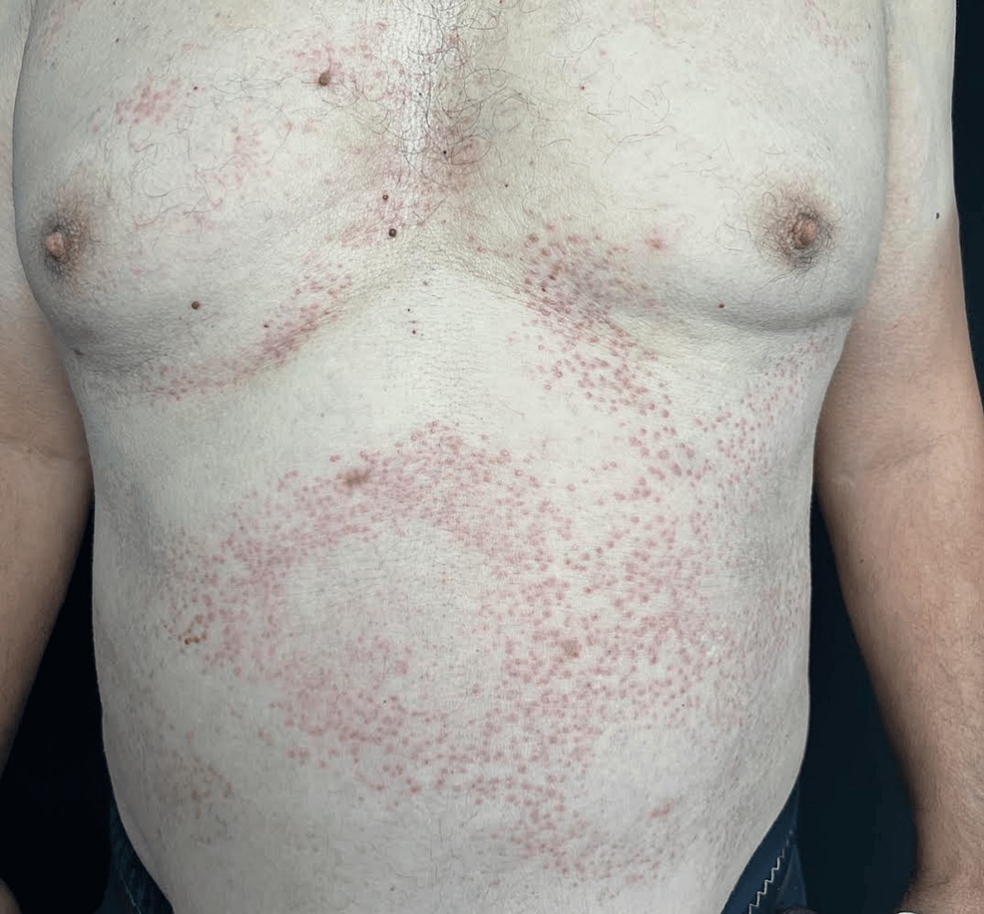
Widespread hyperkeratotic erythematous papules on the chest and abdomen.

**Figure 2:**
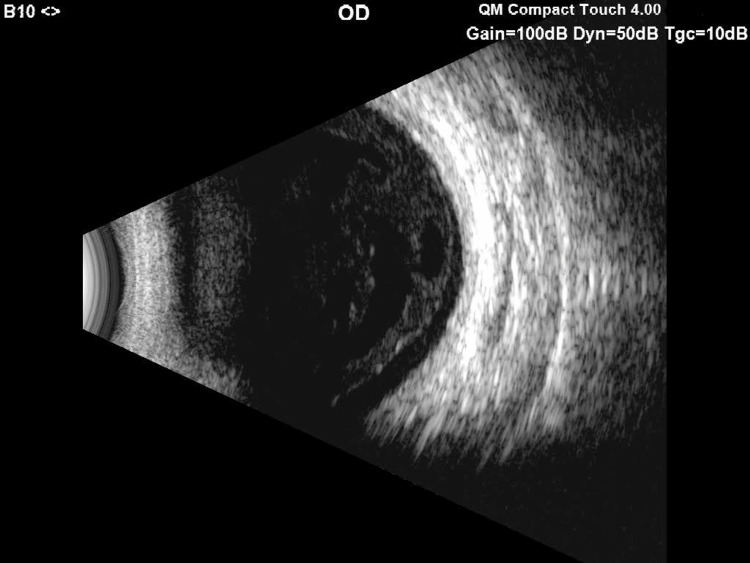
Acute anterior uveitis of the right eye.

A 60-year-old male patient was referred from the Ophthalmology Department for a well-demarcated eruption consisting of asymptomatic, widespread erythematous papules involving the trunk (Figure 1). The patient reported that the lesions had appeared three years before the visit. Shortly afterward, he experienced an episode of acute uveitis (Figure 2). Consequently, the patient was admitted to the Ophthalmology Department, and systemic corticosteroid therapy was initiated. Until the current visit, there have been multiple episodes of acute uveitis accompanied by flares of the skin lesions. Treatment with steroids improved both ocular and dermatological manifestations but was insufficient in controlling subsequent recurrences. Following the exclusion of any local ocular causes, the patient was referred to the Dermatology Department for further investigations. A series of tests were conducted, including a complete blood count, erythrocyte sedimentation rate, C-reactive protein, liver function tests, blood urea, creatinine, glucose, electrolytes, serum calcium, and an electrocardiogram (ECG), all within normal limits. The antinuclear antibody assessment essay was also within normal ranges.

**Figure 3:**
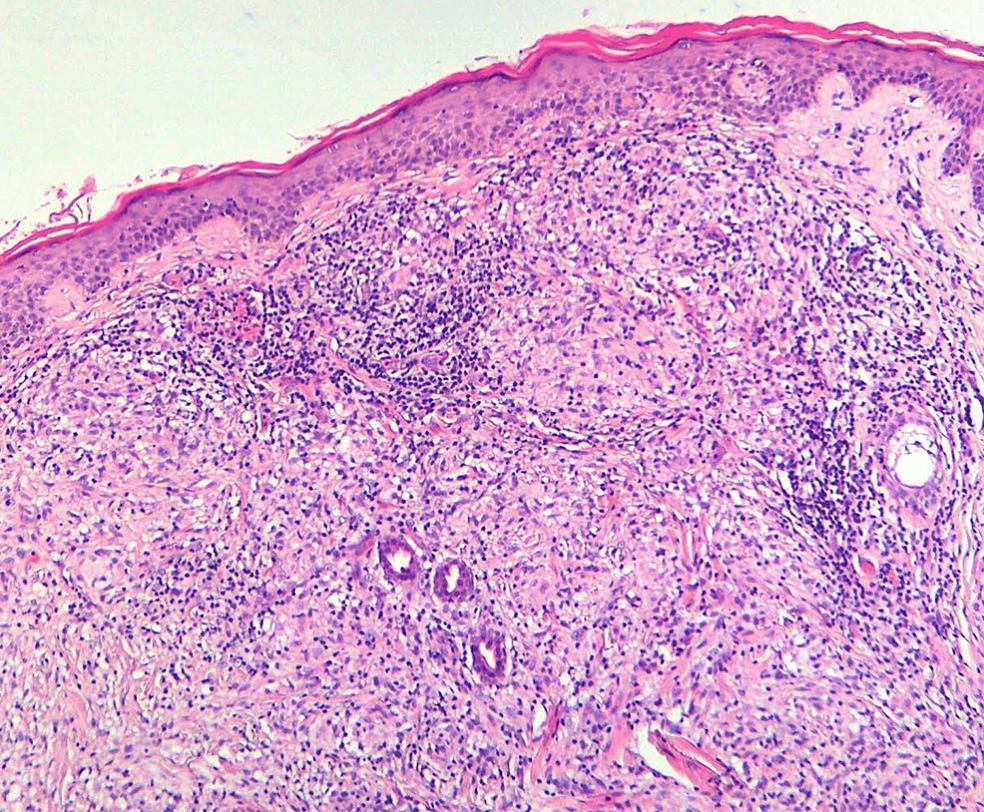
Granuloma annulare: histopathology from a skin biopsy from the abdomen showing orthokeratosis, parakeratosis, lymphohistiocytic granulomas, and degenerated collagen bundles.

**Figure 4:**
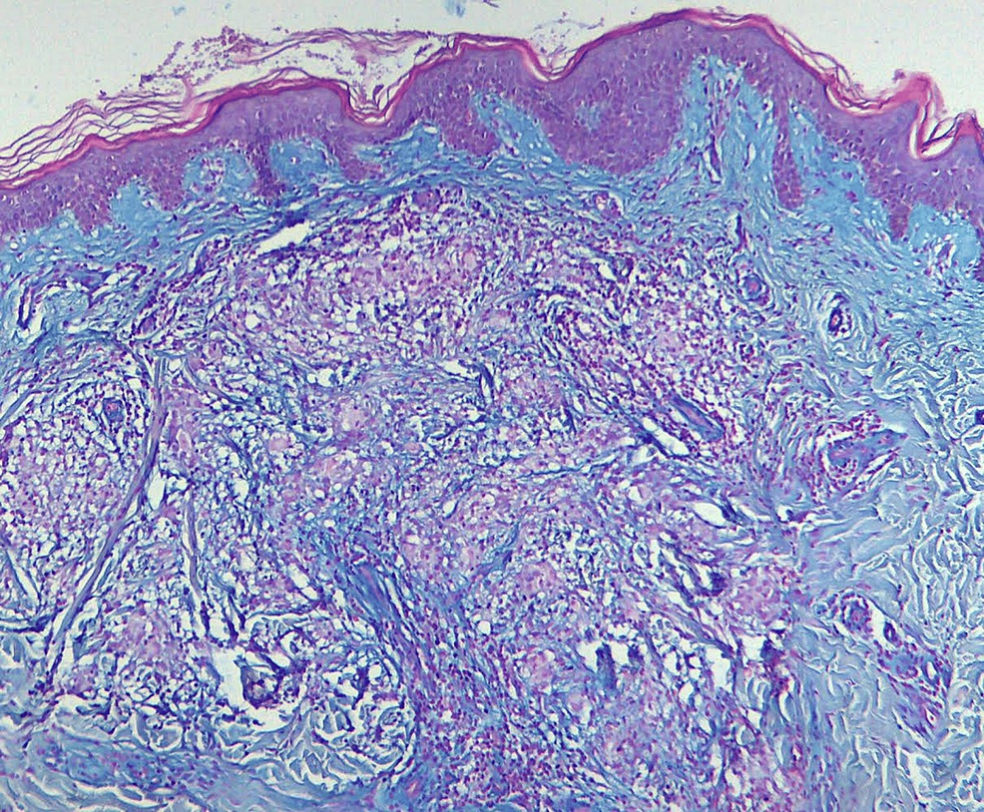
Granuloma annulare: histopathology from a skin biopsy from the abdomen identifying mucin deposition (Alcian blue staining).

**Figure 5:**
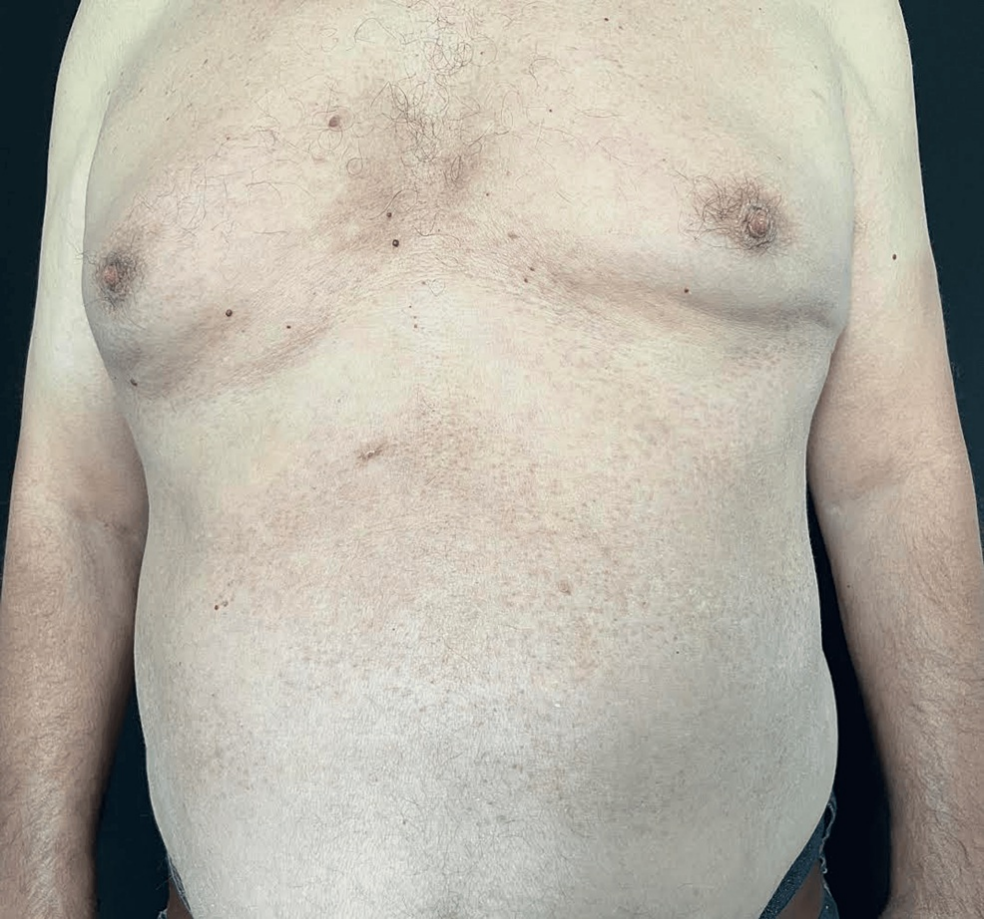
Improvement of chest and abdomen lesions after three months of oral isotretinoin therapy.

The patient presented a medical history of diabetes mellitus type II that was treated with metformin, an oral hypoglycemic agent, at a dosage of 500 mg twice daily. Subsequently, a skin lesion biopsy was performed, and the histopathologic report revealed ortho- and parakeratosis, lymphohistiocytic granulomas surrounding degenerated collagen bundles, and mucin deposition identified through Alcian blue staining (Figures 3, 4). The patient was diagnosed with generalized GA, and therapy with oral isotretinoin 0.5 mg/kg per day was initiated. In the following three months, a positive dermatological outcome was observed (Figure 5). However, a new episode of uveitis occurred. The patient was treated with systemic corticosteroids (prednisone 0.5 mg/kg per day), which were gradually tapered over one month with the resolution of the lesions. A follow-up conducted at three months showed no recurrence of uveitis or GA.

Given the rare association of GA with uveitis and the unanticipated evolution, close monitoring is recommended.

## Discussion

Disseminated GA is a rare and idiopathic skin disease characterized by the formation of annular plaques on the skin. The association between uveitis and GA is rare and has been a subject of interest in the medical literature. Uveitis is an intraocular inflammatory condition primarily involving the uvea (i.e., iris, ciliary body, and choroid) and adjacent structures [8]. It typically affects the anterior uvea but varies in onset, course, severity, and location within the eye [8]. It is believed to be triggered by a broad spectrum of infectious, autoimmune, neoplastic, and traumatic disorders [9].

In a study conducted by Oz et al., a 51-year-old female patient was diagnosed with GA and uveitis simultaneously. The patient presented with symptoms of blurred vision and erythematous, annular papules on the lower and upper extremities. Initially, the patient was treated with topical and systemic corticosteroids, which proved successful. However, after the completion of the therapy, the patient experienced a relapse of the ocular and skin lesions after two months. Systemic corticosteroids were re-administered, leading to regression of the lesions after four weeks [9].

A study by Rahimi and Moinfar reported a case of GA and anterior uveitis treated with topical and systemic corticosteroids. However, relapses were observed once the dose was tapered [10]. Another study by van Kooij et al. revealed that out of eight patients with uveitis and GA, seven developed severe retinal vasculitis [11].

In a study conducted by Brey et al., the significance of regular eye examination for uveitis in patients with GA was investigated. Out of 19 patients who were part of the cross-sectional study, only one developed chronic anterior uveitis. However, since there was a considerable time gap between the onset of the two conditions, this study could not establish any link between them [8].

The treatment of generalized GA remains a challenge. Although topical therapies may improve lesions of localized GA, the daily application of topical corticosteroids to widespread skin lesions can be difficult. Thus, systemic therapy is considered preferable for generalized GA. Several systemic drugs, such as hydroxychloroquine, isotretinoin, or dapsone, have been found to be effective. The selection of these agents is based on the patient's comorbidities and the safety profile of the medication. Alternatively, narrowband ultraviolet B phototherapy can be used as an initial therapy for patients who prefer to avoid systemic treatment.

In the case of our patient, the appearance of recurrent uveitis and the ocular toxicity of hydroxychloroquine led to the proposal of isotretinoin as the initial therapy. Isotretinoin was administered at a daily dose of 0.5 mg/kg and was well-tolerated by the patient.

## Conclusions

GA is a rare, granulomatous skin disease that affects both adults and children. GA is regarded as benign and generally presents with highly variable clinical features. Despite its benign nature, the disease can have a significant impact on patients' quality of life due to its chronicity and unpredictable course. The etiology of GA remains unknown, and the condition is diagnosed based on clinical and histopathological criteria. Treatment options for GA are limited, and management of the disease is generally aimed at alleviating symptoms and reducing the duration of the illness.

The occurrence of GA in conjunction with recurrent uveitis is a rare phenomenon. This association has been observed in only a few cases, and the exact mechanism that links the two conditions is not yet fully understood. More research is needed to determine the implications of this finding and whether regular eye screenings should be conducted for uveitis in patients with GA. Further larger, multicenter, prospective studies are necessary to better understand this association and its potential clinical impact.
